# The 678 Hz acoustic immittance probe tone: a more definitive indicator of PET than the traditional 226 Hz method

**DOI:** 10.1186/s40463-018-0290-y

**Published:** 2018-07-03

**Authors:** Justin M. Pyne, Tarek Ibrahim Lawen, Duncan D. Floyd, Manohar Bance

**Affiliations:** 1 Faculty of Medicine, Dalhousie University, Halifax, NS Canada; 2 Department of Surgery, Division of Otolaryngology – Head and Neck Surgery, Dalhousie University, Faculty of Medicine, Halifax, NS Canada; 3 Department of Clinical Neurosciences, University of Cambridge, Cambridge, UK

## Abstract

**Background:**

The accurate diagnosis of Eustachian tube (ET) dysfunction can be very difficult. Our aim is to determine whether a 678 Hz probe tone is a more accurate indicator of Patulous ET (PET) than the 226 Hz probe tone when used in compliance over time (COT) testing.

**Methods:**

Twenty subjects (11 normal ET ears and 7 PET ears) were individually seated in an examination room and connected to a GSI TympStar Middle Ear Analyzer. The order of probe tone frequency (678 or 226 Hz) was randomized. Baseline “testing” COT recordings for each ear undergoing testing were completed. Subjects were instructed to occlude their contralateral nostril and to breathe forcefully in and out through their ipsilateral nostril until the test had run to completion. This process was repeated with the probe tone that had not been previously run. For the control group, each subject had one random ear tested. For the experimental group, only the affected ear(s) was tested. Wilcoxon rank rum tests were performed to determine statistical significance.

**Results:**

The baseline COT measurements for the control group and PET group were similar, 0.86 mL (SD = 0.34) and 0.74 (SD = 0.33) respectively. Comparing the 226 Hz tone between groups revealed that PET patients had a median COT difference 0.19 mL higher than healthy ET patients, and for the 678 Hz tone, PET patients had a median COT difference of 0.57 mL higher than healthy ET patients. Both were deemed to be statistically significant (*p* = 0.002, *p* = 0.004 respectively). The was a statistically significant median COT difference between the 678 Hz and 226 Hz of 0.61 mL (*p* = 0.034) for the PET group, while the same comparison for the control group of 0.05 mL was not significant (*p* = 0.262), suggesting that the 678 Hz tone yields a larger response for PET than the 226 Hz tone, and no difference for the control group, thus making it less prone to artifact noise interference.

**Conclusion:**

The 678 Hz probe tone is a more reliable indicator of ET patency, and should be preferably used over the 226 Hz tone for future COT testing.

## Background

The Eustachian tube (ET) is a narrow, epithelial-lined osseocartilaginous tube that connects the middle ear cavity to the nasopharynx [1, 2]. It functions to maintain middle ear health and to facilitate sound transmission from the tympanic membrane (TM) to the inner ear [3]. The ET accomplishes this by fulfilling three major physiologic roles, which include drainage of middle ear secretions, prevention of nasopharyngeal reflux and, most importantly, pressure equalization across the TM [4, 5]. The proper functioning of the ET greatly depends on the regular intermittent opening and closing of the tube. At rest, the ET is passively collapsed; however, the ET can be actively opened under the control of paratubal muscles during such activities as swallowing, yawning and chewing [2, 6]. Any aberration in opening and closing is considered ET dysfunction (ETD) and can be further classified as either obstructive or patulous dysfunction [3, 4, 7].

Obstructive ETD is inadequate tubal opening caused by either paratubal muscular failure or obstruction of the ET by intrinsic changes. Failure of the ET to open typically results in patients complaining of aural fullness, periodic ‘popping’ sounds, muffled hearing and tinnitus [8]. Left untreated, patients with obstructive ETD can develop cholesteatoma, perforation, middle ear effusions and conductive hearing loss [4, 9].

A patulous Eustachian tube (PET) is a far less common type of ETD where the ET remains abnormally open intermittently or permanently, allowing for excessive communication between the middle ear and nasopharynx [10]. Reported potential risk factors for PET include sudden and severe weight loss, pregnancy, radiation therapy and congenital ET defects; nonetheless, many patients do not have any predisposing factors [10–16].

The most typical symptoms of PET include autophony (hearing one’s own voice) and aerophony (hearing one’s own breathing), and often aural fullness [17]. Vertigo, tinnitus and conductive hearing loss have been described, but in our experience, are less common [18]. In severe cases, symptoms are so distressing that patients progress to develop psychiatric sequelae, such as suicidal ideation and major depressive episodes [15]. Some symptoms, such as autophony can overlap with other disorders, such as superior canal dehiscence. Classically, autophony and aerophony are made worse with standing and exercise, and are improved by lying down [19]. However, symptoms can be intermittent and not present when the patient is seen in clinic. The definitive diagnosis of PET is made on direct otoscopic observation of the tympanic membrane moving synchronously with respiration: this is the result of transmission of nasopharyngeal air pressures directly to the middle ear cavity (MEC) through the patent ET. The movement of the tympanic membrane can be exacerbated by having the subject sit upright and take deep breaths while occluding one nostril, to accentuate nasopharyngeal pressure changes [19].

Despite several different methods being employed over the years, there is no universally accepted protocol for the evaluation of a patent ET [10]. Nasal endoscopy is frequently used to examine the pharyngeal opening of the ET; however, due to a narrow tubular lumen, the opening being eccentric to the line of visualization, the valve area being mostly hidden, and the presence of secretions, diagnostic visualization of the ET is usually very difficult [20].

Various researchers have suggested the use of acoustic immittance and standard tympanometry to indirectly observe the respiratory-synchronous movements of the tympanic membrane in the presence of PET [4, 21, 22].

During an acoustic immitance measurement, the tympanometer generates a pure tone of a specific frequency that is delivered into the ear canal via its probe component., and the reflected sound measured. This is a measure of the acoustic impedance and admittance. The typical, tympanometric probe tone is 226 Hz. Acoustic admittance (the reciprocal of acoustic impedance) is a reliable surrogate for compliance when the tone is of a relatively low frequency.

A tympanometer can graph the compliance over time (COT) of the tympanic membrane (sometimes called long time-base tympanometry, and often found on the reflex decay testing function in commercial tympanometers), and this is helpful in the evaluation ET function. During the test, the patient is asked to perform various breathing exercises (e.g. ipsilateral nostril breathing or sniffing) to see the effect on the tympanic membrane’s COT. Exaggerated changes in tympanic membrane compliance synchronous with inhalation and exhalation are indicative of a PET [10].

Compliance over time using 226 Hz pure tones has been shown to identify PET via respiratory-synchronous middle ear compliance with occlusion of the contralateral nostril and forced ipsilateral nostril breathing [10, 22–24]. Nonetheless, we have found in our ET practice that alterations in COT measurements using a 678 Hz pure tone is more powerfully predictive of PET than any other tone used before. This was observed when using COT testing with the 226 Hz frequency in the Eustachian Tube clinic; anecdotally, the false-positive rate was too high and the signals too small to reliably interpret with the 226 Hz tone.

The aim of this study was to evaluate results of COT testing in presumed closed and patulous Eustachian tubes, and to compare the 226 Hz and 678 Hz probe tones to determine if the latter provides a clearer distinction between patulous ET and closed ET, which has not been previously described. We hypothesize, based on our observations, that the PET group will produce stronger, clearer, and more identifiable patterns of COT using a 678 Hz pure tone when compared with the 226 Hz frequency, while the closed ET group will yield similar results for each respective tone.

## Methods

### Subject selection

Ethics approval was obtained from our institutional research ethics board. Through the Eustachian Tube Clinic at our institution, subjects were identified as having suspected PET and asked to participate in this study. These subjects experienced one or more of the symptoms of autophony, aerophony, and/or aural fullness. All PET subjects were examined with otoscopy and microscopy to confirm that there was no obstruction in the ear canal, no current visible ear disease, and that the TM was moving with respiration prior to testing. Any subjects not meeting these criteria were excluded from the PET group (experimental group). These were our confirmed PET subjects.

To obtain our healthy ET group (control group), subjects with no prior history of ear disease, no previous ear surgery, and no current ET dysfunction were recruited. All healthy subjects were examined with otoscopy to confirm that there was no obstruction in the ear canal, no current visible ear disease, and that the TM was not moving with respiration prior to testing. Any ears not meeting these criteria were excluded from the control group.

### Data collection

Data was collected prospectively. Subjects were seated upright in an examination room and connected to a GSI TympStar Middle Ear Analyzer (Grason-Stadler, MN, USA). A typical tympanogram was completed on each subject. The machine was then set to the acoustic reflex decay test protocol (allowing for a 15-s recording/10 s analysis window) and either the 226 Hz probe tone or the 678 Hz probe tone was selected. The acoustic reflex stimulus setting was set to the CONTRA option, but the contralateral earphone was not placed in the subject’s opposite ear; the stimulus level was also set to 35 dB, i.e. there was no acoustic stimulus applied that could cause a stapedial reflex. The order of probe tone frequency (678 or 226 Hz) was randomized. Baseline “testing” COT recordings for each ear undergoing testing were completed. This measured the middle ear admittance over the 15 s window in the absence of any acoustic stimuli or subject maneuvering. As such, this was the admittance over time with the middle ear “at rest.” COT testing for PET was then completed. Just prior to initiating the test, each subject was instructed to occlude their contralateral nostril. The testing protocol was initiated, and each subject was instructed breathe forcefully in and out through their ipsilateral nostril until the test had run to completion, which increases nasopharyngeal pressure changes, and therefore emphasize the effects of a PET. This process was repeated with the probe tone that had not been previously run. For the control group, each subject had one random ear tested. For the experimental group, only the affected ear(s) was tested. All results were retrieved from the TympStar printer upon completion of each test run.

### Statistical analysis

A descriptive analysis was performed on patient demographics. These are represented by averages, range, and standard deviation. For statistical analysis, COT values at both 226 Hz and 678 Hz were compared between healthy and PET subjects, as well as within each subject group. A case control design was implemented. With PET patients representing cases and healthy ET patients representing controls. Wilcoxon rank sum tests were performed to determine statistical significance. All analysis was performed with R version 3.3.1 (“Bug in Your Hair”). Effect sizes are presented as medians and approximate 95% confidence intervals. Non-parametric testing was utilized as sample sizes in our study were insufficient to rely on assumptions of asymptomatic normality for the purposes of hypothesis testing. A *p* value < 0.05 was considered statistically significant (95% confidence interval.)

### Study population

The control group consisted of eight males and three females with a mean age of 28.7 years (range: 24 years to 46 years; SD = 5.8 years). Of this group, three members described having symptoms of tinnitus intermittently. None had hearing loss or experienced autophony.

The experimental group consisted of four males and three females with a mean age of 35.7 years (range: 23 years to 50 years; SD = 12.3 years). Of these patients, two had a sensorineural hearing loss and one had a hearing loss that was conductive in nature. Three patients from the experimental group could volitionally open their ET, and tests were performed when they were sure their ETs were open, which was confirmed by observation of movement of the TM with respiration. Four patients reported symptoms of autophony. None reported tinnitus. Data are reported as means and standard deviations (SD).

## Results

The study included a total of 18 patients (18 ears) who underwent COT testing, demographics are in Table 1. Both the average and range of magnitude of MEC were recorded for each group.

**Table 1:** Demographics of study population

	Mean Age, yrs (range)	Male	Female
Healthy ET	28.63 (24–46)	8	3
PET	35.71 (22–50)	4	3

**Fig. 1:**
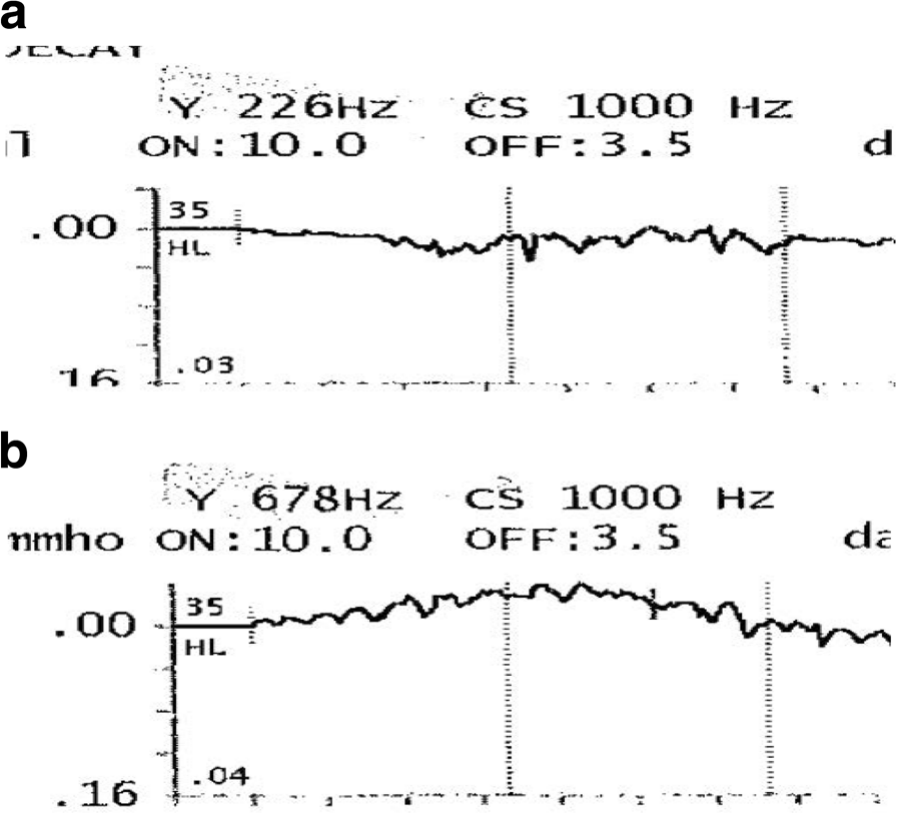
**a** and **b** Tympanogram tracings of a healthy ET subject during testing with 226 Hz (**a**) and 678 Hz (**b**) probe tone. The y-axis represents middle ear compliance (MEC), measured in mL or mmho. The x-axis represents time, measured in seconds (s). A detailed outline of the procedure is described in the “Data Collection” section of this manuscript. Based on an algorithm used by the creators of the TympStar, the maximum MEC during testing is calculated. The tracings indicate movement of the TM with each respiration

**Fig.2:**
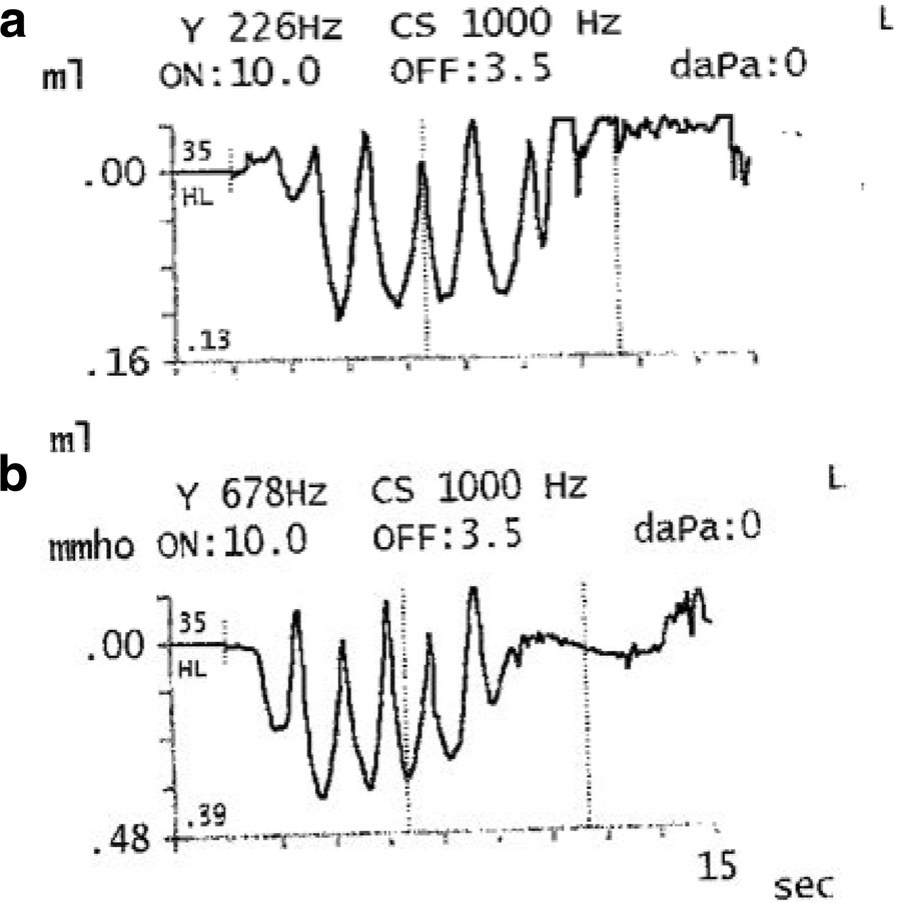
**a** and **b** Tympanogram tracings of a PET subject during testing with 226 Hz (**a**) and 678 Hz (**b**) probe tone. The y-axis represents middle ear compliance (MEC), measured in mL or mmho. The x-axis represents time, measured in seconds (s). A detailed outline of the procedure is described in the “Data Collection” section of this manuscript. Based on an algorithm used by the creators of the TympStar, the maximum MEC during testing is calculated. The tracings indicate movement of the TM with each respiration

In the control group (11 ears), the resting tympanogram compliance average was 0.86 mL (SD = 0.34 mL), which is comparable to that of the experimental group (0.74 mL, SD = 0.33 mL), however two PET ears were missed for this stage of testing. The average change in middle ear compliance (COT) was 0.07 mL (SD = 0.05 mL) for the 226 Hz frequency, and 0.12 mL (SD = 0.12 mL) for the 678 Hz frequency (Table 2). Figure 1 shows COT tracings for a healthy ET subject at the 226 Hz and 678 Hz frequencies. For the PET group (7 ears), the average middle ear compliance change was 0.26 mL (SD = 0.08 mL) and 0.69 mL (SD = 0.35 mL) for the 226 Hz and 678 Hz frequencies, respectively (Table 2). Figure 2 shows COT tracings for a PET subject at the 226 Hz and 678 Hz frequencies.

**Table 2:** Comparison of healthy ET and PET COT following testing of 226 Hz and 678 Hz probe tones

	226 Hz		678 Hz	
	Average COT (mL)	SD (mL)	Average COT (mL)	SD (mL)
Healthy ET	0.07	0.05	0.12	0.12
PET	0.26	0.08	0.69	0.35

**Fig. 3:**
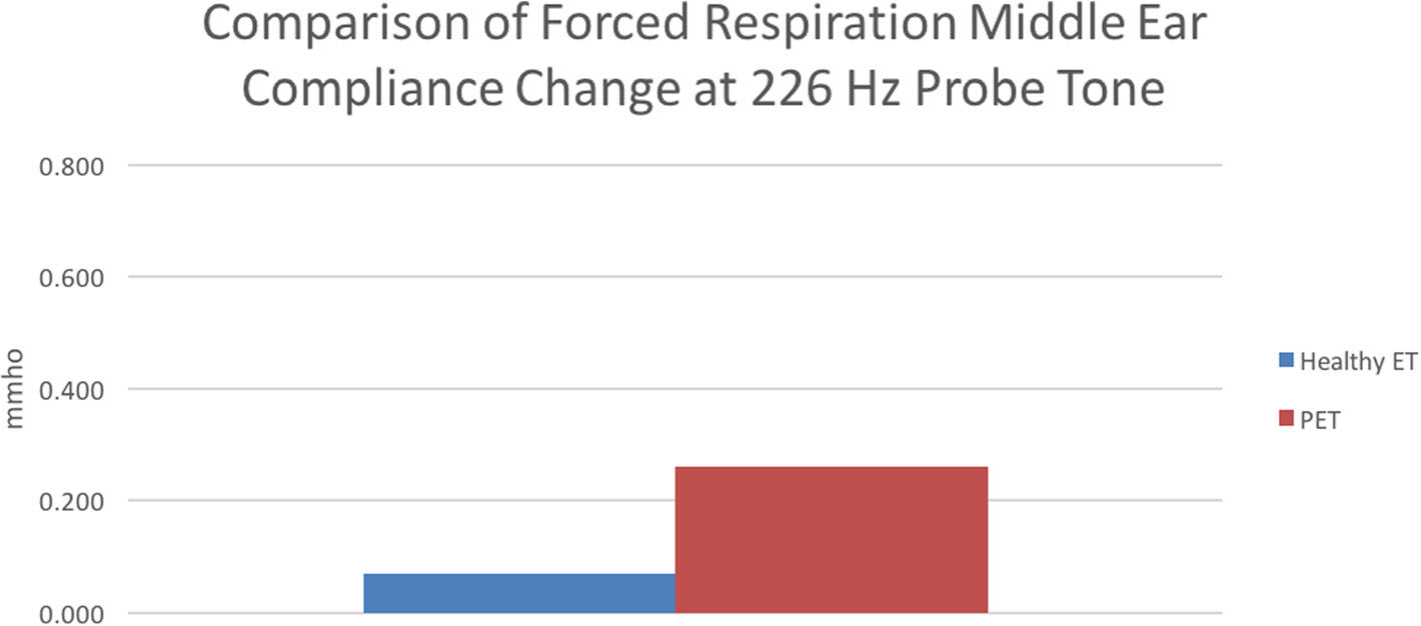
Comparison of middle ear compliance (MEC) between healthy ET and PET subjects at the 226 Hz probe tone frequency. The y-axis represents MEC, measured in mmho. The x-axis displays each respective group, with healthy ET represented by the blue bar, and PET represented by the red bar. It was found that the PET group had significantly higher MEC than the healthy ET group during testing with forced respiration (*p* = 0.02)

**Fig. 4:**
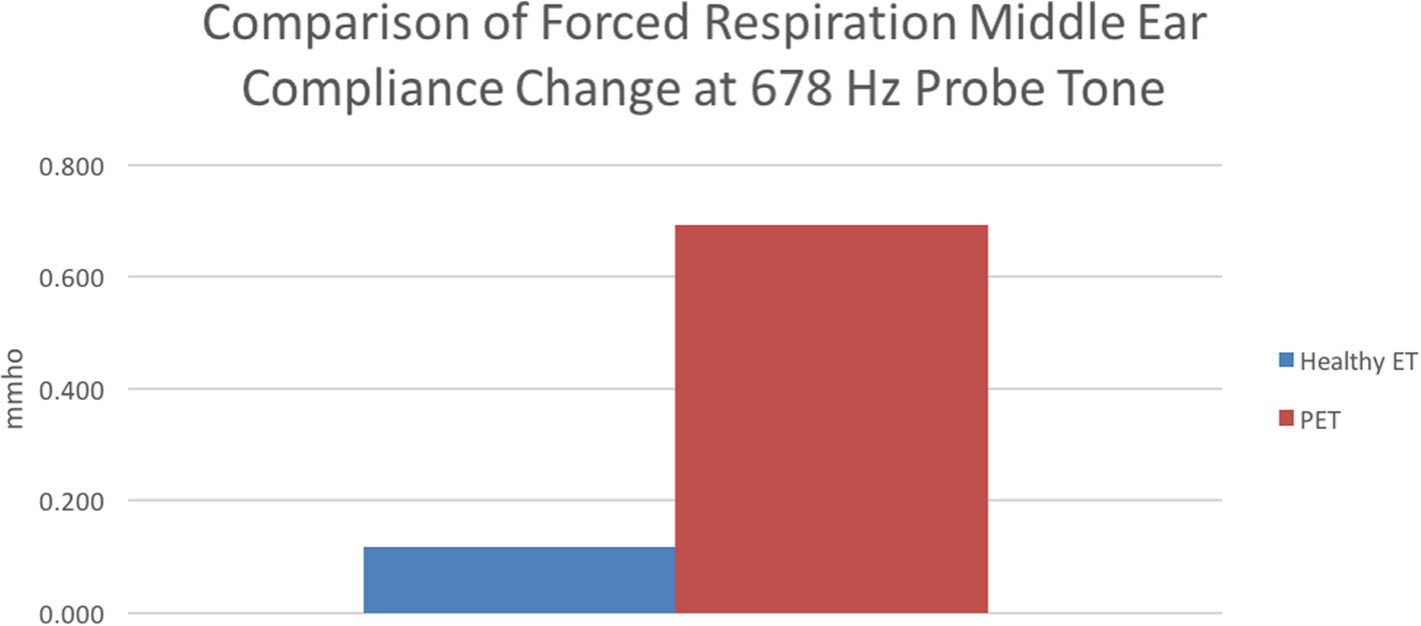
Comparison of middle ear compliance (MEC) between healthy ET and PET subjects at the 678 Hz probe tone frequency. The y-axis represents MEC, measured in mmho. The x-axis displays each respective group, with healthy ET represented by the blue bar, and PET represented by the red bar. It was found that the PET group had significantly higher MEC than the healthy ET group during testing with forced respiration (*p* = 0.04)

For comparison of results between groups, the difference between median COT at each frequency was evaluated (Table 3). It was found that for the 226 Hz frequency, the experimental group had an average COT of 0.19 mL more than the control group (0.26 mL vs 0.07 mL) and this was statistically significant (*p* = 0.002, Fig. 3). The median COT difference between groups at the 678 Hz frequency the difference was found to be 0.57 mL (0.69 mL vs. 0.12 mL) and this statistically significant (*p* = 0.004, Fig. 4). The difference between COT at 678 Hz and 226 Hz for the control group was 0.05 mL, while for the experimental group, this difference was 0.61 mL. The later was found to be statistically significant (*p* = 0.034) while there was no significant relationship seen in the former (*p* = 0.262).

**Table 3:** Wilcoxon Rank-Sum test for COT difference between healthy ET and PET at 226 Hz and 678 Hz probe tones

	Median Difference	95 CI	p
PET 226 Hz vs. healthy ET 226 Hz	0.19	(0.09 to 0.28)	0.002
PET 678 Hz vs. healthy ET 678 Hz	0.57	(0.25 to 0.93)	0.004
Healthy 678 Hz vs Healthy 226 Hz	0.05	(−0.02 to 0.09)	0.262
PET 678 Hz vs PET 226 Hz	0.61	(0.01 to 0.79)	0.034

## Discussion

Evaluation of ET function via tympanometry has been used for many years, but it certainly has its challenges. Primarily, the 226 Hz tone provides only limited reliability for distinguishing between healthy and diseased ET states. This study investigated the compliance over time (COT) of patulous ET and healthy ET patients when subjected to 226 Hz and 678 Hz tones, with the goal of determining if the latter yielded a clearer distinction between the healthy and disease ET states. Although based on our statistical analysis, both the 226 Hz and 678 Hz frequencies yielded results that indicate they can distinguish between healthy ET and PET states, in comparison with the former, the latter method shows a very strong response for PET subjects, and in some cases, the response was higher than the limits of the TympStar instrument. This limitation is due to the fact the we have independently adapted this machine to run the tests we have set up. As such, the TympStar as commercially configured is not the ideal instrument for measuring positive PET responses. Nonetheless, the upper limit of recording was 0.99 mmho and any response that exceeded this value were recorded as the maximum (0.99 mmho). If an instrument with the capability of reading up to 2.0 mmho or even 3.0 mmho was available, a more exact measurement of MEC would be possible. While the 226 Hz frequency does appear to show a distinction between healthy ET and PET states, the response is much lower than the 678 Hz probe tone, suggesting that the former could yield false positive results due to random noise.

One interesting aspect of our analysis was the comparison of probe tones within each subject group. There was no statistically significant difference between the 226 Hz and 678 Hz frequencies COT responses for the control group, which would support the notion that both are equally as effective at avoiding a false positive non- healthy ET state. However, comparing the same two tones for the experimental group, we found that the 678 Hz frequency had a significantly higher response than the 226 Hz frequency, offering further support that the former is the ideal frequency for this methodology of testing.

When evaluating the readouts from the instrument, it appeared that there was an apparent discrepancy between the displayed reading and the paper tracing produced. According to the manufacturer of the TympStar machine, a complicated and proprietary algorithm – which is beyond the scope of this paper – is used to produce the peak value reading, and little weight should be given to the tracings per se, rather the actual numbers reported on screen are more accurate.

It is important to acknowledge one of the limitations to our study: the sample size. However, given that many subjects with intermittent PET displayed no eardrum movements at the time of testing, or they stopped between microscopic examination and going to the testing area, it is difficult to obtain a large population of gold standard subjects. Thus, a longer study period may afford the necessary amount of time to discover a larger group of subjects.

## Conclusion

To our knowledge, this is the first study to describe the 678 Hz probe tone in evaluation of middle ear immitance and compliance over time. Based on the evidence presented here, this suggests that this tone is a more reliable distinguisher between healthy ET and PET states than the previously accepted frequency. A study aimed at a larger population would be ideal for further evaluation. Additionally, using an instrument with the ability to record larger amplitude responses would allow more precise and accurate measurements of PET responses.

## Abbreviations

ET: Eustachian Tube
ETD: Eustachian Tube dysfunction
MEC: Middle ear cavity
PET: Patulous Eustachian tube
TM: Tympanic membrane
